# A Low-Cost Non-explosive Synthesis of Graphene Oxide for Scalable Applications

**DOI:** 10.1038/s41598-018-30613-4

**Published:** 2018-08-13

**Authors:** Pranay Ranjan, Shweta Agrawal, Apurva Sinha, T. Rajagopala Rao, Jayakumar Balakrishnan, Ajay D. Thakur

**Affiliations:** 1 Department of Physics, Indian Institute of Technology Patna, Bihta, 801106 India; 2 Department of Chemistry, Indian Institute of Technology Patna, Bihta, 801106 India; 3 Department of Physics, Indian Institute of Technology Palakkad, Palakkad, 678557 India

## Abstract

A low cost, non-explosive process for the synthesis of graphene oxide (GO) is demonstrated. Using suitable choice of reaction parameters including temperature and time, this recipe does not require expensive membranes for filtration of carbonaceous and metallic residues. A pre-cooling protocol is introduced to control the explosive nature of the highly exothermic reactions during the oxidation process. This alleviates the requirement for expensive membranes and completely eliminates the explosive nature of intermediate reaction steps when compared to existing methods. High quality of the synthesized GO is corroborated using a host of characterization techniques including X-ray diffraction, optical spectroscopy, X-ray photoemission spectroscopy and current-voltage characteristics. Simple reduction protocol using ultra-violet light is demonstrated for potential application in the area of photovoltaics. Using different reduction protocols together with the proposed inexpensive method, reduced GO samples with tunable conductance over a wide range of values is demonstrated. Density functional theory is employed to understand the structure of GO. We anticipate that this scalable approach will catalyze large scale applications of GO.

## Introduction

Graphene oxide (GO), a monolayer sheet of graphite oxide, is by no means a less wonderful material than graphene^1–33^. It not only serves as a precursor for making reduced graphene oxide (rGO) but also itself has remarkable physical and chemical properties which makes it a sought after material for applications in areas which include electronics, biomedicine, energy and environment^1–25^. GO has promising biological applications demonstrated at laboratory scale which include, drug delivery^1,2^, antibacterial coatings^3–5^, photo-thermal cancer therapy^6,7^ and selective differentiation of mesenchymal^8^ and neural^9,10^ stem cells. Key environmental applications of GO include contaminant adsorption^11,12^, water decontamination^13–15^, solar desalination^16^ and environmental sensing^17,18^. GO also finds niche applications in areas including tribology^19,20^ and energy storage^21,22,28^. GO has been shown to be a highly flexible nanomaterial with a high stiffness^23^. Furthermore, the recent demonstration of high quality graphene prepared using microwave reduction of GO^24^ has the potential to open the flood-gates for real world electronic applications of GO. However, realizing all this in practice hinges on developing a safe, economic and scalable method for making GO.

Chemical routes based on oxidative-exfoliation methods have been developed over the years for the synthesis of GO^34–56^. Table S1 provides a summary of these synthesis protocols along with their environmental impact and cost. Historically, the first report on GO is credited to Brodie who in 1859 treated graphite with a strong oxidizing mixture of potassium chlorate (KClO_3_) and fuming nitric acid (HNO_3_) to obtain a derivative which he named ‘oxide de graphite’^34^. In a series of efforts that followed, Gottschalk^35^ confirmed Brodie’s findings and Staundenmaier worked on improving the yield and safety of the reactions involved^36^ with limited success. Staundenmaier primarily attempted replacing fuming HNO_3_ by a mixture of sulphuric acid (H_2_SO_4_) and fuming HNO_3_^36^. Other significant early reports include those by Berthelot^37^, Luzi^38^, Charpy^39^, Hamdy^40^, Weinschenk^41^ and by Kohlschütter and Haenni^42^, however, with little success in controlling the explosive nature of the reactions involved. A new era in GO research was unleashed with the usage of powder X-ray diffraction for a better understanding of the structure^43–46,48^ and magic angle spinning nuclear magnetic resonance (MAS-NMR) for investigating the nature of functional groups^49^. Hummers and Offeman^50^ got rid of the requirement for KClO_3_ and HNO_3_ using a reaction which involved gradual addition of potassium permanganate (KMnO_4_) to a suspension of powdered graphite and sodium nitrate (NaNO_3_) in H_2_SO_4_ (Hummers’ method). This considerably reduces the explosive nature of the reaction but a major issue remains due to the emission of toxic gases including NO_2_ and N_2_O_4_. Furthermore, contamination due to Mn residues has also been a major hurdle in getting high quality GO. Several groups used various modifications to the Hummers’ approach however, the issues related to contamination by Mn residues, toxic gas emission and the explosive nature of the reactions were not completely eliminated^51,52^. A further modification to Hummers’ method was suggested by Marcano *et al*.^53^ where they claimed absence of any Mn residues and in addition there were no toxic gas emissions. Although their synthesis starts with the use of inexpensive graphite powder, Marcano *et al*.^53^ recommended extensive and cumbersome washing, filtration, centrifugation and dialysis steps. These involved the use of expensive membranes. Using simple but key modifications to synthesis parameters, we propose a method for Mn residue free GO synthesis which does not involve the use of expensive membranes without compromising on the quality of the yield. Furthermore our synthesis technique completely eliminates the explosive nature of reaction by following a set of fixed protocols unlike methods reported earlier in the literature^50,52–54^. To corroborate the high quality of the yield, we characterized the as synthesized GO using a host of techniques including X-ray diffraction, Raman spectroscopy, UV-Visible spectroscopy, Fourier transform infra-red spectroscopy, electron microscopy and X-ray photoemission spectroscopy. We show typical applications which include: (a) diodic response across the aluminum zinc oxide (AZO)-GO multi-layer, and (b) response to 1.5 AM Global sunlight of a GO-rGO multi-layer demonstrating the the photovoltaic phenomena. The rGO film for (b) above was prepared by a simple UV-light reduction^57,58^ of GO film made using doctor-blade technique^59^. In addition, we also utilize a host of reduction techniques^24,57,58,60,61^ to demonstrate the preparation of rGO using GO synthesized using our approach with tunable conductance over a wide range of values. Furthermore, GO foam (which has a host of possible applications)^1–4,6–8,10^ can be easily prepared following our approach. We anticipate that our inexpensive scalable synthesis approach will catalyze large scale applications of GO.

## Results and Discussions

**Figure 1:**
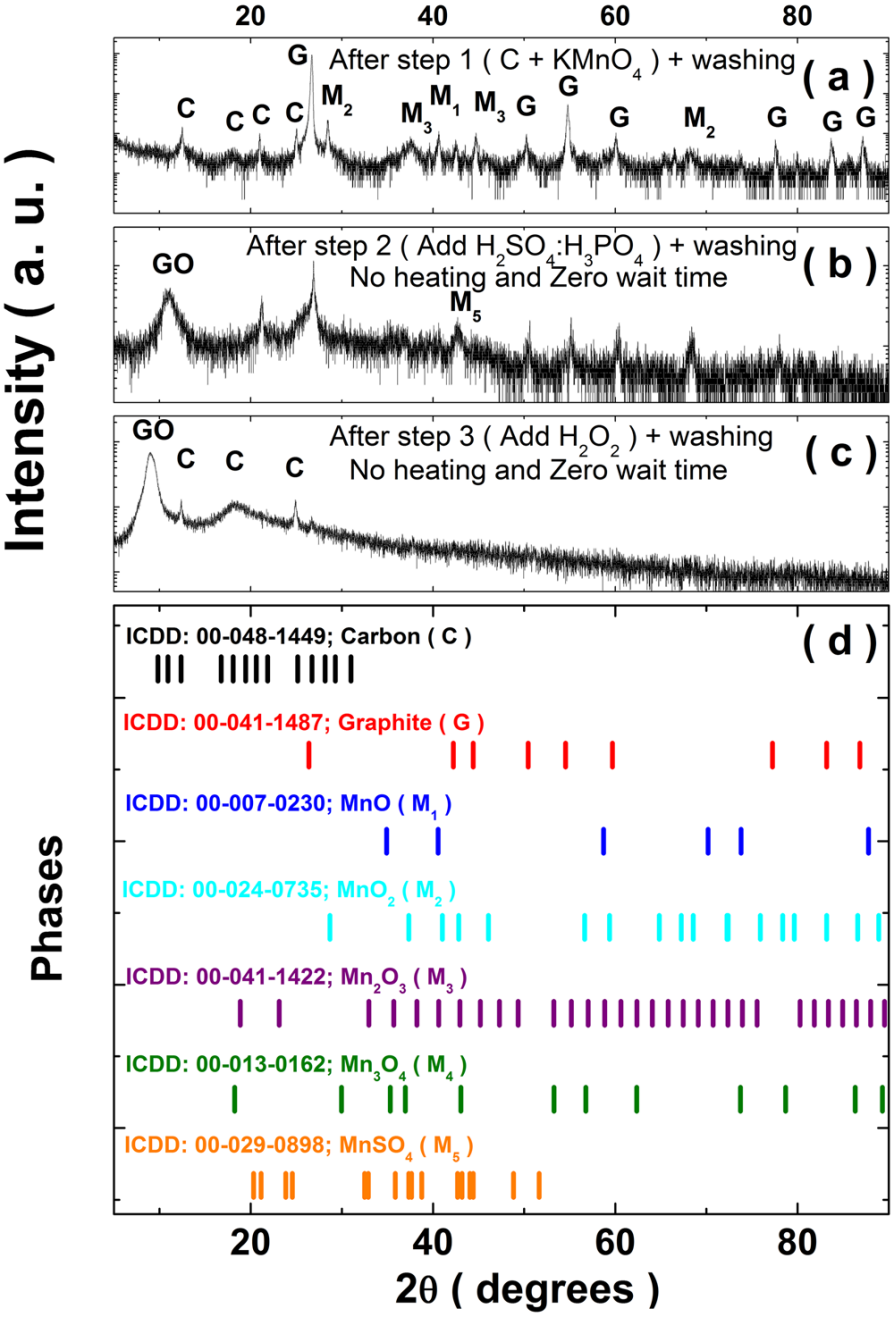
XRD spectra of (**a**) graphite (C) and potassium permanganate (KMnO_4_) mixture after washing, (**b**) Samples in (**a**) mixed with sulphuric acid and hydrogen peroxide (without heating) after washing and with zero soaking time, (**c**) Sample in (**b**) with heating and washing, (**d**) ICDD card number of carbon, graphite and various Mn oxides.

It is evident from literature^34,36,50,52–54^ that the major impediments during synthesis of GO include: (a) explosive nature of the underlying reactions, and (b) existence of graphitic and Mn-based residues in the final product. In order to tackle these problems, it is necessary to understand the reaction intermediaries at each stage of the reaction process which comprises of steps corresponding to intercalation, exfoliation and termination of reaction^34,36,50,52–54^. Such information bear tremendous potential for addressing the reason for presence of graphitic and Mn-based residues in GO. At the outset, we implemented a pre-cooling protocol (PCP) to control the highly exothermic nature of the involved reactions which to us appeared as the primary reason for its explosive nature. The PCP comprises of individually pre-cooling the initial reaction mixtures of: (a) C:KMnO_4_, and (b) H_2_SO_4_:H_3_PO_4_ to an initial temperature of 5 °C. This simple protocol ensures that the temperatures do not rise beyond room temperatures irrespective of the method^54^ by which the acid mixture is added to C:KMnO_4_ mixture. Thus, PCP completely eliminates any possibility of explosions during the reaction. For a better understanding of the reaction progress, we have truncated the reaction at each stage and looked at the washed product. In particular, we have looked at the reaction products after: (a) mixing graphite (C) and KMnO_4_ (designated as step-1), (b)adding H_2_SO_4_:H_3_PO_4_ solution to the C:KMnO_4_ mixture (designated as step-2), and (c) addition of H_2_O_2_ post step-2 (designated as step-3). This was done without any further heating and soaking time after step-2 as well as with various soaking times (in the range of 0–24 h) at several temperatures (in the range of 20–65 °C) after step-2. XRD data was obtained for the resulting samples at the end of step-1, step-2 and step-3, respectively and the results for zero soaking time are summarized in Fig. 1(a–c). For a comparison, the stick diagrams corresponding to the diffraction patterns for graphite, rhombohedral carbon, various oxides of Mn (MnO, MnO_2_, Mn_2_O_3_ and Mn_3_O_4_) and MnSO_4_ are provided in Fig. 1(d). After step-1, we observe the presence of XRD peaks corresponding to various oxides of Mn in addition to those for graphite and rhombohedral carbon. After step-2, the hints for the initiation of the formation of GO is evident in the form of a broad peak centered at 11 °C. However, despite the washing step, the peaks corresponding to various Mn oxides and MnSO_4_ phase are still present after step-2. There are distinct color changes at the end of each step. After step-1, the color is black and it changes to greenish black after step-2. As mentioned earlier, the temperature of the reacting mixture does not rise beyond room temperature during steps 1 to 3. However, after step-2, there is a distinct change in color from greenish black to dark brown if the reaction mixture is heated above room temperature. Upon addition of H_2_O_2_ in step-3 we observe a sudden change in color from dark brown to golden yellow which is a visual marker for the formation of GO^33^. We observed that unlike Marcano *et al*.^53^, the required amount of H_2_O_2_ was considerably higher in our method (almost twice the concentration of H_2_O_2_ in H_2_O:H_2_O_2_ solution). The XRD of the washed sample obtained after step-3 shows a prominent diffraction peak corresponding to GO. Here, we don’t observe any prominent peak corresponding to Mn oxides, however, the peaks corresponding to rhombohedral carbon are still present. Next, we look at the XRD patterns for the samples prepared with different soaking times and temperatures, as shown in Fig. S2 (see SI). From this exercise, we conclude that good quality GO samples can be synthesized following steps 1 to 3 with a soaking time of 24 hours at 65 °C. The GO samples so prepared donot have any Mn residues and spurious carbonaceous residues without the need for any sophisticated filtration protocol.

**Figure 2:**
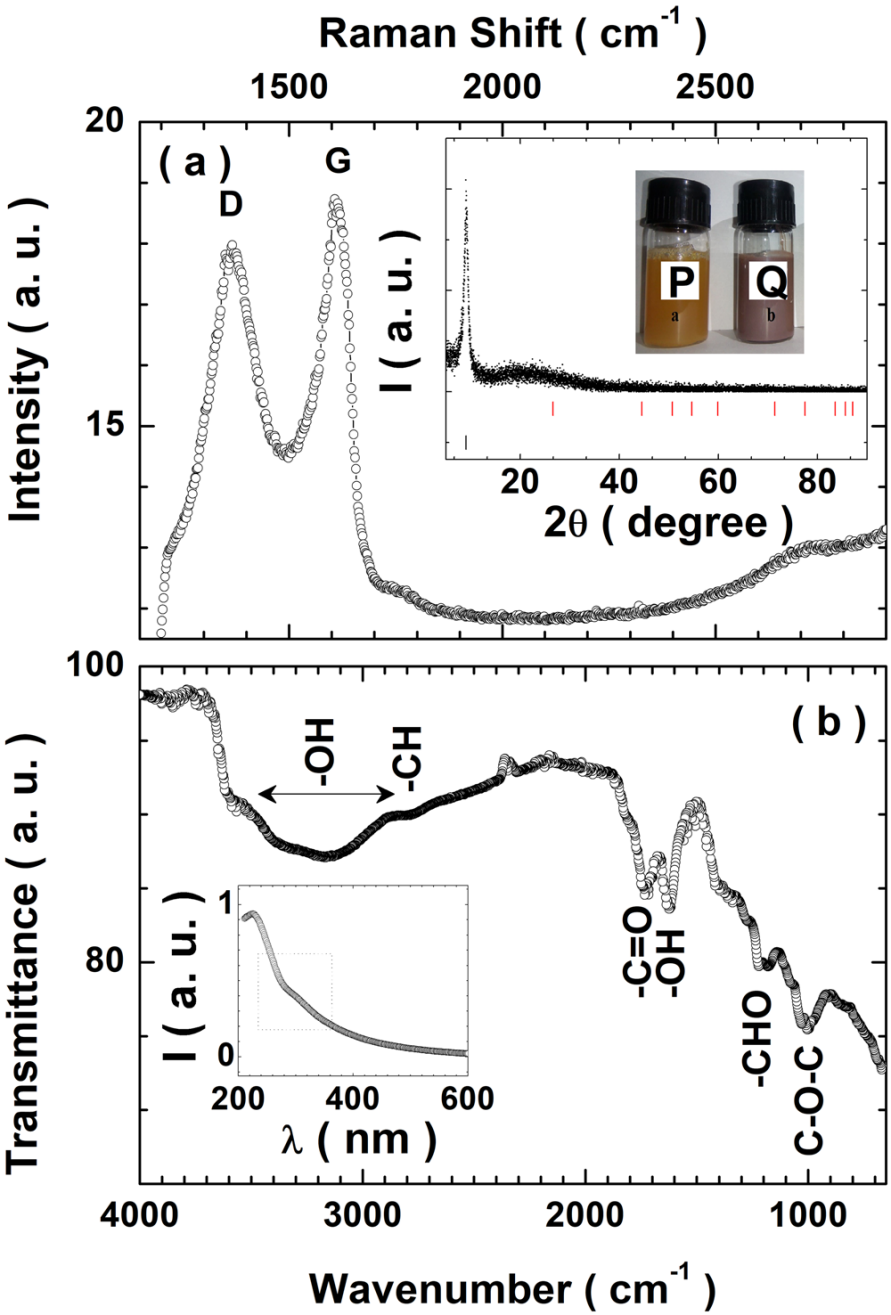
(**a**) Raman spectra for the drop casted film of the as grown GO sample. The D and G peaks are marked. The slight hump in the wavenumber range 2550–2780 cm^−1^ corresponds to the 2D peak. The inset in panel (a) shows the powder x-ray diffraction data for the dried as grown sample. It shows the characteristic peak corresponding to GO. The bottle marked P shows the dispersed GO sample whereas the bottle marked Q contains the reaction mixture just prior to adding H_2_O_2_ to terminate the reaction used in our process. (**b**) FTIR spectra for the dried as grown GO sample obtained in the transmittance mode. The bands and dips corresponding to various functional groups are marked. The inset in panel (b) shows the UV-Vis spectra for the as grown GO sample dispersed in de-ionised water. The region enclosed by the square (dotted lines) is characteristic feature corresponding to GO.

Figure 2(a) shows the Raman spectra of the GO film obtained at room temperature in the wavenumber range 1200–2900 cm^−1^. The D-band corresponding to 1350 cm^−1^ and the G-band corresponding to 1580 cm^−1^ are marked. In addition, a wide hump at the high wavenumber end corresponding to the 2D band is also observed. The G-band originates due to: (i) the bond stretching of *sp*^2^ hybridised *C*=*C* in both rings and chains, and (ii) first order scattering from the doubly generated E_2*g*_ phonon modes in the Brillouin zone centre. The G-band therefore contains information on the *sp*^2^ hybridised carbon network. On the other hand, the D-band is associated with the breathing mode of the aromatic rings which is caused by the structural imperfections due to the attachment of oxygen based functional groups in the carbon basal plane. Therefore, D-band is a measure of the degree of defects. The *I*_*D*_/*I*_*G*_ ratio of 1.1 indicates the presence of structural defects within the as synthesized sample^33,62^. The presence of 2D band centered around 2680 cm^−1^ can be attributed to the double resonance transitions and is also an overtone of the D band. The inset in panel (a) of Fig. 2 shows the Intensity (I) versus 2 *θ* plot obtained from the X-ray diffraction (XRD) of the as synthesized GO sample. For a comparison, the stick diagrams for the XRD corresponding to graphite and GO from the International crystallography diffraction database (ICDD) are shown. The interlayer spacing of GO provides crucial information on the degree of intercalation of various oxide functional groups during oxidation. We observe a diffraction peak at 2 *θ* = 9.11°corresponding to an interlayer spacing of 9.74 Å which indicates a very high degree of intercalation^53,56^. Table S1 (see SI) compares the interlayer spacing along with details of the synthesis procedures reported by various groups. The absence of the prominent graphitic peak at 26.5° (corresponding to the interlayer spacing of 3.35 Å) and the appearance of the graphene peak at 9.11° is a fingerprint for the formation of GO^63,64^. Sub inset in panel (a) shows the optical image of the bottle marked P which contains dispersed GO sample whereas the bottle marked Q contains optical image of the reaction mixture just prior to adding H_2_O_2_. The inset in panel (b) of Fig. 2 shows the UV-Vis spectrum of GO dispersed in water. It consists of a peak at 233 nm and a broad shoulder between 285–305 nm. The 233 nm peak corresponds to the *π*-π^*^ transition of *C*=*C* bonds while the broad shoulder can be attributed to the *n*-π^*^ transition of the *C*=*O* bonds^33,53,56,65,66^. The combined information from the methodology used in preparing the samples and the corresponding XRD data, UV-Vis spectra and the Raman shift data is hallmark for the high degree of intercalation of oxide functional groups. The Fourier transform infra-red (FTIR) spectroscopy is capable of elucidating the nature of various functional groups in the GO sample. Figure 2(b) shows the FTIR transmittance spectra of the as synthesized GO sample. The broad peak observed in the wavenumber range of 2900–3500 cm^−1^ can be attributed to the stretching mode of the ***O*-*H*** groups. The ***O*-*H*** groups in GO are bonded to the parent carbon network at various locations (ranging from the center of the sheet to its borders). The corresponding shifts in the frequency of vibration of the ***O*-*H*** bonds lead to a resultant broadening of the band. Presence of residual water molecules intercalated between the GO sheets also contributes to the broadening of the ***O*-*H*** band. The strong band centered at 1725 cm^−1^ can be attributed to the stretching vibration of *C*=*O* bonds in carboxyl/carbonyl groups. The band at 1616 cm^−1^ can be attributed to the vibration of ***O*-*H*** groups in water molecules adsorbed on GO sheets. Similarly, the bands at 1224 cm^−1^ and 1055 cm^−1^ can be attributed to the stretching vibrations of the ***C*–*OH*** group and the *C*–*O* (epoxy) group respectively^67–69^. There is no significant band at ≈1570 cm^−1^ (corresponding to the stretching vibrations within graphitic domains) pointing to a high quality of the as synthesized GO sample.

**Figure 3:**
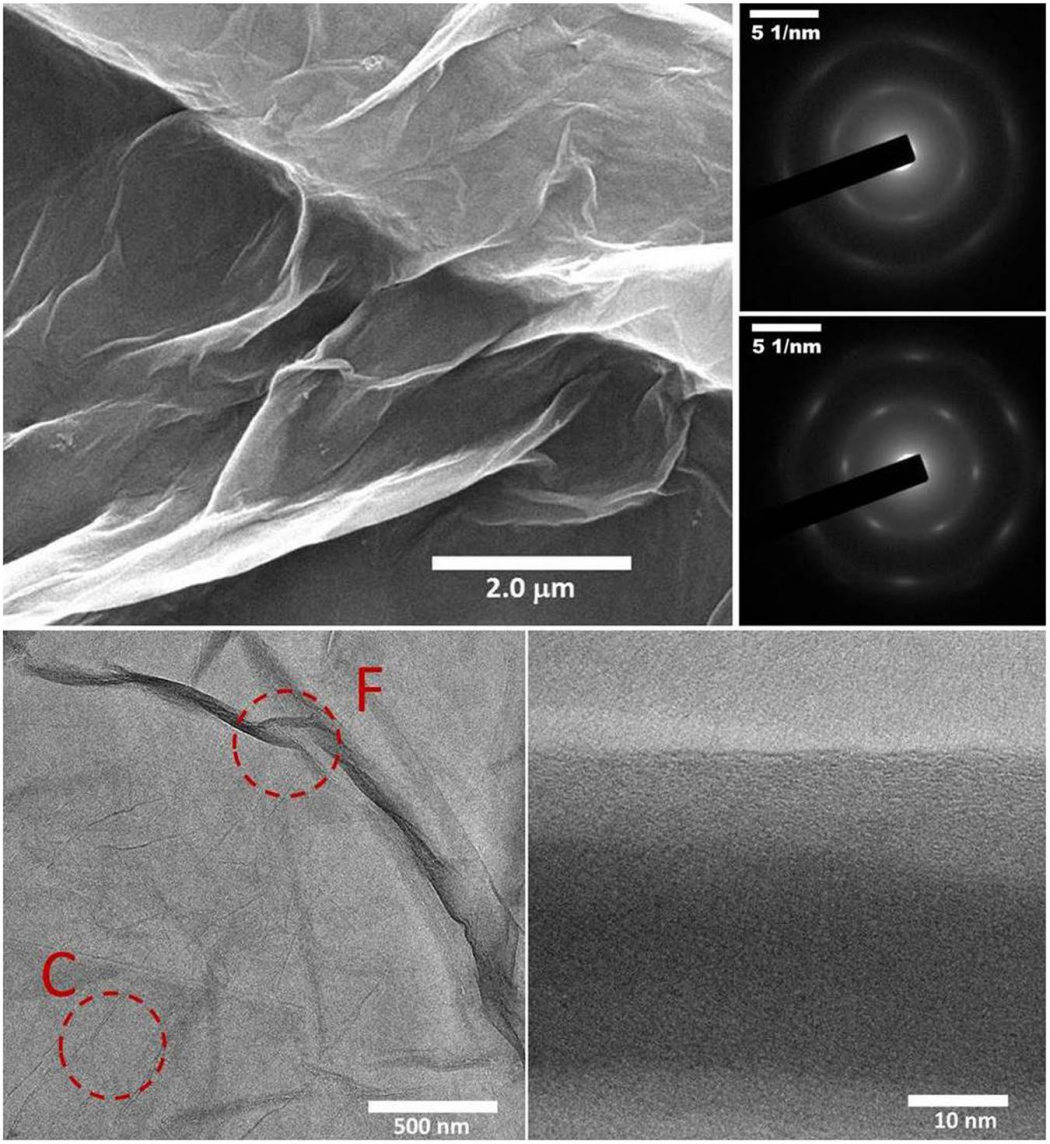
(**a**) SEM image of a spin coated GO film. (**b**) TEM image of a drop casted GO film on the TEM grid. The regions F and C are marked corresponding to the region with and without the wrinkles/folds. (**c**) TEM image of a portion of the drop casted film containing a fold/wrinkle. (**d**) SAED pattern obtained from a region containing wrinkles/folds (similar to F marked in panel (b)). (**e**) SAED pattern obtained from a region which does not contain any wrinkles/folds (similar to C marked in panel (b)).

The surface morphology of the synthesized GO sheets is studied using a scanning electron microscope (SEM). The SEM image of a portion of the GO sheet is shown in Fig. 3(a). We observe uniform sheets of GO over micron scales. Certain wrinkles and folds are also observed^70^. Transmission electron microscopy (TEM) has the potential to elucidate the details related to crystallinity in addition to morphology of nanomaterials and has been extensively used to study GO sheets^53,56,71–73^. We perform TEM characterization of the as synthesized GO sheets. Panels (b) and (c) of Fig. 3 show the TEM images at different magnifications. Regions F and C are marked in Fig. 3(b) where a fold is present and absent, respectively. In particular, Fig. 3(c) shows a TEM image across a fold in the GO sheet. Absence of any visible substructure across the fold suggests the high quality GO nanosheets. Panels (d) and (e) of Fig. 3 shows the selective area diffraction (SAED) patterns obtained in regions F and C, respectively. The six-fold spots observed in the SAED patterns obtained from both of the regions F and C points to the underlying honeycomb graphene like skeleton. A comparison of the two SAED patterns suggests the existence of a diffuse ring like structure along with less sharp six-fold spots in the region F. This indicates the far greater disorder in the carbon skeleton underneath the folds.

**Figure 4:**
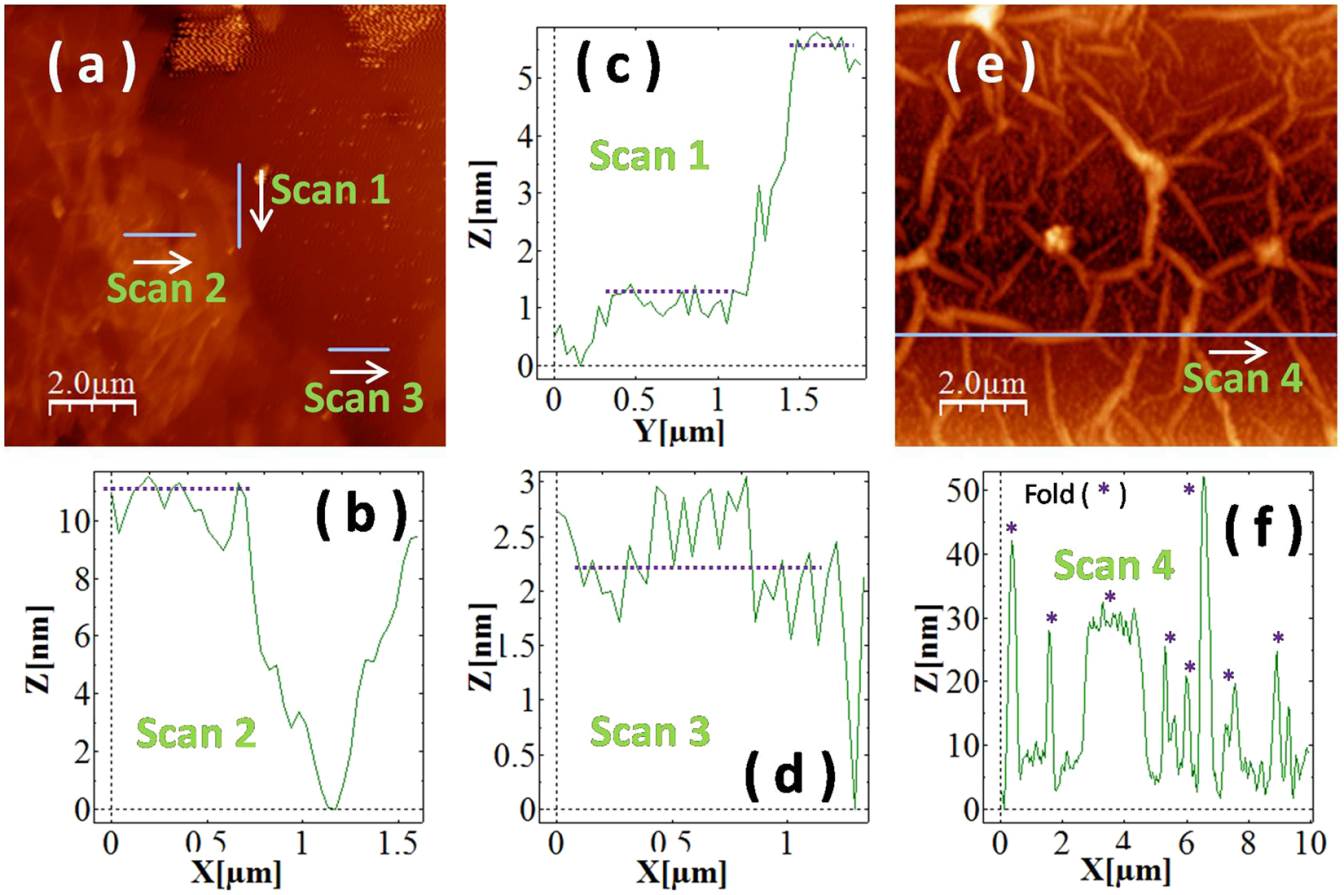
(**a**) AFM image of a drop coated GO film. (**b**), (**c**) and (**d**) height profile data of scan2, scan1 and scan3 respectively in (**a**). (**e**) AFM image of drop casted GO film with corrugation and wrinkles. (**f**) Height profile of the GO film along the wrinkles obtained in (**e**).

In its pristine form, GO comprises a single atomic layer thick graphene-like carbon sheet (GLCS) with oxygen containing functional groups (OCFGs) extending out of the plane at several locations on both faces. The OCFGs are covalently bonded to the carbon atoms transforming them to the sp^3^ hybridized state from their sp^2^ hybridized state in the GLCS. This leads to the existence of oxidized domains in the midst of GLCS domains. The number density of OCFGs increases with the degree of oxidation. A pristine GO flake truly exists only in the solution phase (in water or in low molecular weight alcohols) where it is completely exfoliated into single-layer sheets. When deposited on a substrate (say glass), a GO flake is in contact with the substrate on one side and with air on the on the other side with a significant likelihood of physisorbed water molecules on both sides. The vacuum dried drop-casted film of GO (dispersed in water) on a glass substrate is rough on an atomic scale with thicknesses varying from single layer to several tens of layers. Atomic force microscopy (AFM) is unable to individually resolve the GLCS domains and the OCFG containing domains and provides only an average thickness for the GO flakes. We observe a single layer flake thickness of about 1.1 nm with a lateral extent of about 1–1.2 *μ*m (see scan 1 in Fig. 4(a,c)). Similarly, layered flake thickness ranging from 2–8 nm with lateral dimensions of 0.6–1.2 *μ*m are shown in Fig. 4(b,d). This agrees well with the reports in literature^52,53,56^. On the other hand, a thick GO sheet with a spatial extent of about 10 *μ*m is also seen Fig. 4(e) and corroborates well with the SEM image of the drop-casted film of GO. Prominent wrinkles are also observed across the drop-casted sample as shown in Fig. 4(e). It should be noted that the bulk form of GO (which can be a GO gel with enhanced water content, or, a GO foam with low water content) results from a restacking of the exfoliated single-layered (and multi-layered) GO sheets. This is very different from the cases when GO is dispersed in a solvent.

**Figure 5:**
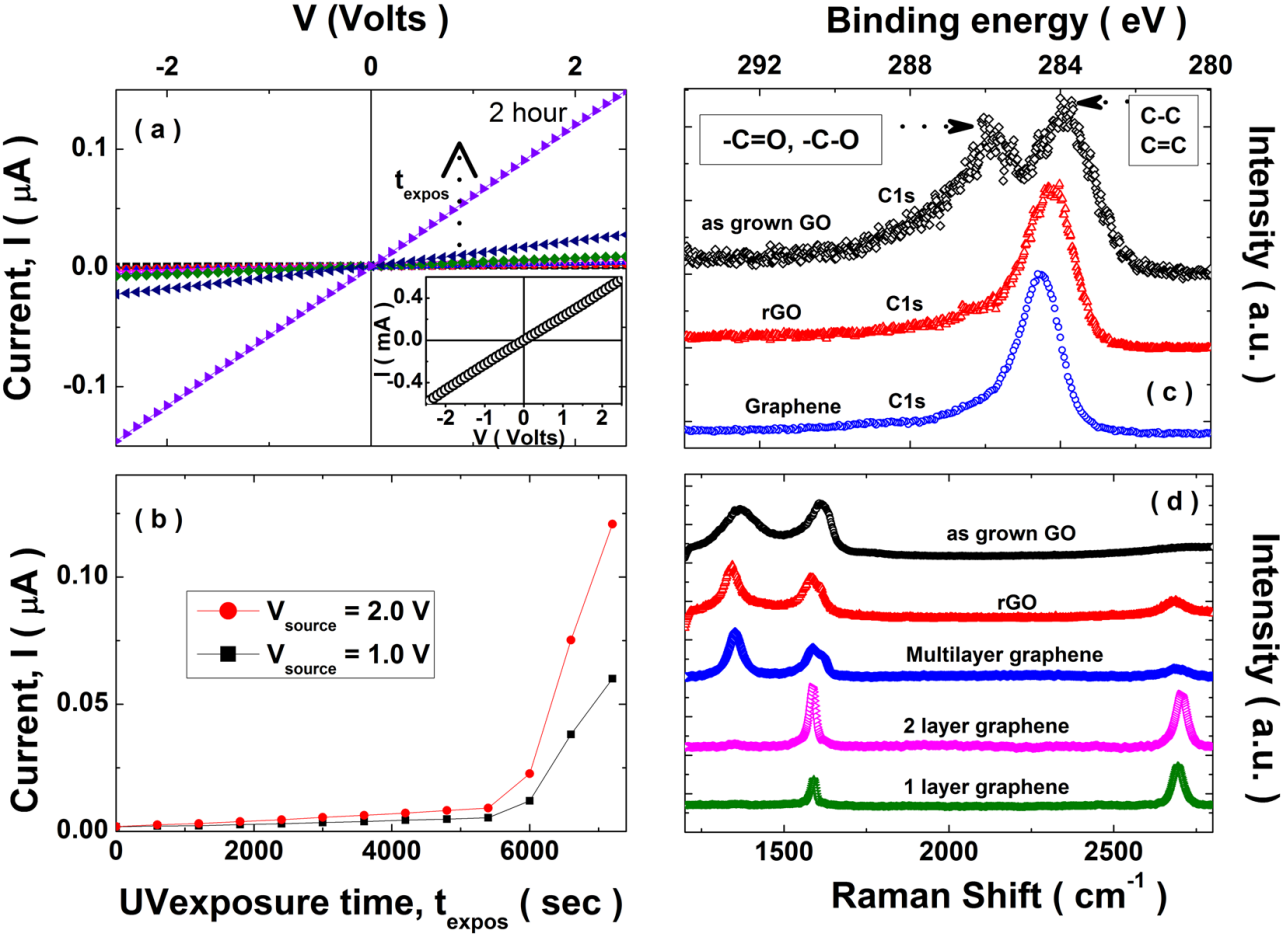
(**a**) The UV exposure dependent current-voltage (I-V) characteristics of a GO film drop-casted on a glass substrate. These I-V characteristics were obtained after different durations of exposure (ranging from 0–2 hour) to a 4 Watt, UV lamp emitting at 365 nm. Inset: I-V characteristics of CVD grown graphene, (**b**) Current (I) as a function of UV exposure time keeping the voltage (V) across the GO film fixed at 1.0 Volt and 2.0 Volt, respectively. (**c**) C 1 s XPS obtained for the GO and the rGO (reduced via exposure to a 4 Watt, 365 nm UV lamp for 2 hours) samples and that of CVD grown graphene. The peaks corresponding to the various functional groups are marked. (**d**) The comparison of Raman spectra for the GO film and the rGO film obtained after a 2 hour exposure to a 4 Watt, 365 nm UV lamp and Raman spectra of 1 L, 2 L and multilayer CVD grown graphene. The D, G and 2D peaks are appropriately labeled.

GO films were deposited on a glass substrate using spin coating. We employed a reduction protocol where a 4 Watt, 365 nm UV lamp was used for exposure of the as synthesized GO films for different periods of time. The current-voltage (I-V) characteristics for the GO films were measured as a function of UV exposure time. For this, we used a four-probe configuration and looked at current across the two inner terminals as a function of the source voltage applied across the two outer terminals. The evolution of the I-V characteristics with the UV exposure time (up to 2 hours) is shown in Fig. 5(a). For a comparison, the I-V characteristic of CVD graphene is shown in the inset of Fig. 5(a). In Fig. 5(b), we plot the increase in current as a function of UV exposure time at typical source voltages of 1 and 2 Volts. A significant increase in current is seen for exposure times beyond 90 minutes (5400 s). To corroborate the reduction of GO to rGO we obtained the Raman and X-ray photoemission (XPS) spectra both prior to UV exposure as well as post 2 hours exposure for samples belonging to the same batch of synthesis. Figure 5(c) shows a comparison of the C1s XPS spectra of the as grown sample, the post 2 hours UV exposed sample and the CVD grown graphene. A significant suppression of the peak(s) corresponding to the oxygen functional groups^32^ is observed in the 2 hour UV exposed sample. It was found that the full width half maxima of rGO sample was 4.73 eV while that for CVD grown graphene was 3.9 eV. The difference in FWHM of the rGO and pristine graphene suggests the presence of residual functional groups in rGO sample that require further reduction to achieve electronic properties similar to that of pristine graphene. In Fig. 5(d), we show a comparison of the Raman spectra of the as grown sample and a corresponding sample after 2 hours exposure to UV light. The characteristic D, G and 2D peaks are labeled. There is a significant increase in the 2D peak with the UV exposure demonstrating the enhancement in the sp^2^ bonded carbons akin to rGO. One can also see a slight increase in the D peak which hints that the reduced sample has a correspondingly higher degree of disorder compared to the parent sample. This together with the used synthesis protocol for GO, demonstrates the feasibility of an inexpensive technique for the preparation of good quality rGO thin films. Moreover, for a comparison of reduced GO Raman spectra with pristine graphene (CVD grown), graphene single layer (I_2*D*_/I_*G*_ =  2.7), bilayer (I_2*D*_/I _*G*_ = 1.4) and multilayer Raman spectra is shown in Fig. 5(d). Based on statistical Raman mapping, Eigler *et al*.^74^ provided a recipe for estimating extent of disorder in rGO. A conservative estimate of the extent of disorder based on the observed *I*_*D*_/*I*_*G*_ ratio of the 2 hour reduced GO sample can be made and it is estimated to be less that 0.06%^74^.

**Figure 6:**
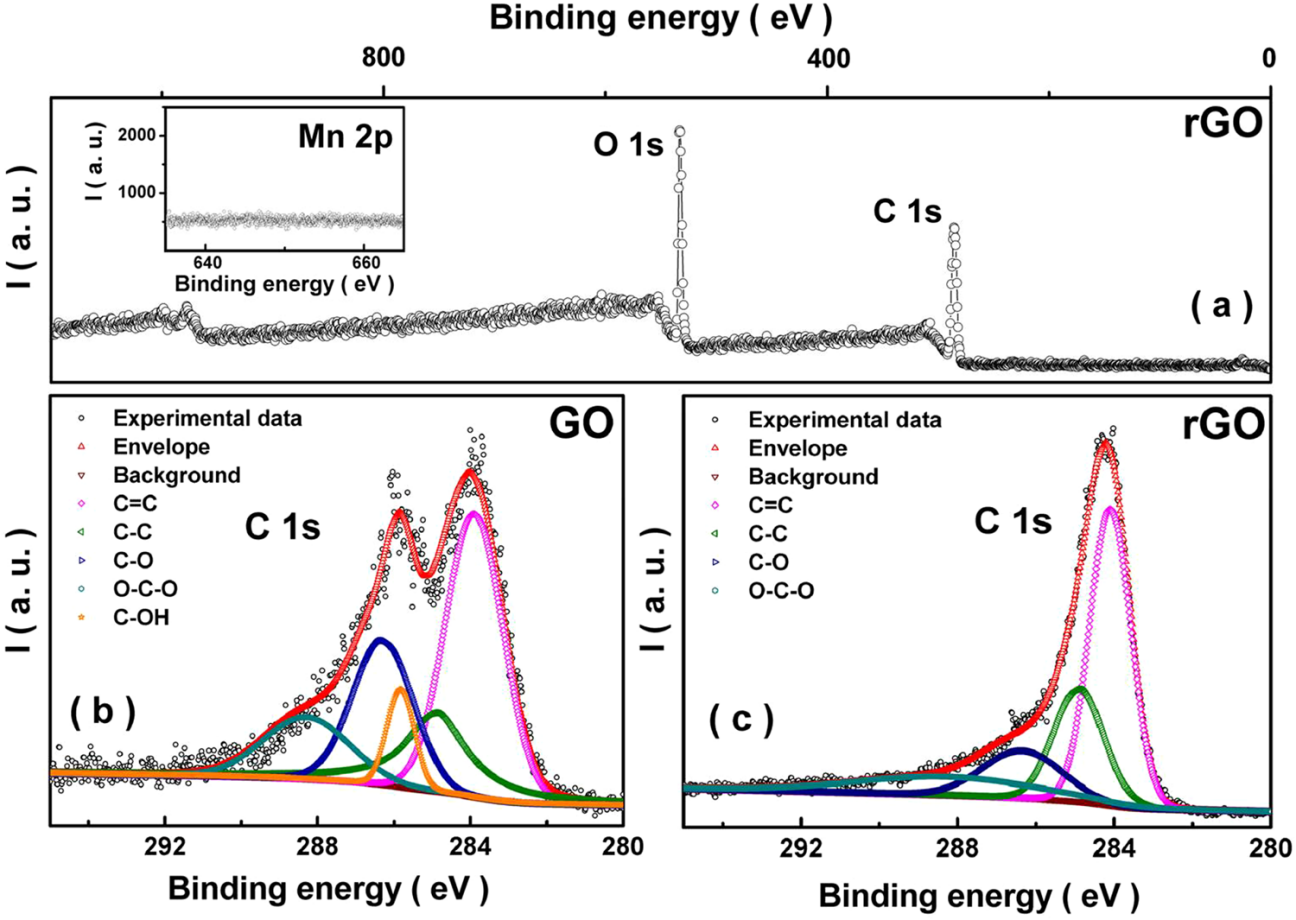
(**a**) Intensity (I) versus binding energy (B. E.) in the full scan range for the XPS of the rGO film obtained after a 2 hour exposure to a 4 Watt, 365 nm UV lamp. (**b**) A multiple peak deconvolution of the GO C1s XPS data (corresponding to –C–C–, –C=C–, –C–O, –O–C–O–, –OH and –C=O respectively). The inset in panel (a) shows the XPS data corresponding to Mn 2p binding energy region for the rGO sample. (**c**) rGO C1s XPS data for the sample along with the deconvoluted peak structure corresponding to –C–C–, –C=C–, –C–O, –O–C–O–, and –C=O are marked.

In order to further ascertain the quality of rGO samples made by us, we performed a careful analysis of the XPS data. Figure 6(a) shows the intensity (I) versus binding energy (B. E.) in the full scan range for the XPS of the rGO film obtained post 2 hour exposure to a 4 Watt, 365 nm UV lamp. One can clearly observe the absence of Mn 2p peak (see inset panel in Fig. 6(b) for further details). It should be noted that there is wide a degree of variability in the detection limits for individual elements using XPS depending on the exact details of the host matrix^75^. For the detection of the presence of Mn residues in GO matrix, it would be safe to assume that the minimum detection limit is about 0.03 at.%^75^. The absence of any observable Mn 2p peak corroborates the absence of Mn residues in the GO samples made using our synthesis process up to these detection limits. A multiple peak fit analysis of the C 1 s of GO and rGO data are shown in Fig. 6(b,c), respectively. Peaks corresponding to –C–C–, –C=C–, –C–O, –C=O, –O–C–O– and –OH are marked appropriately. On comparison of the C1s XPS spectrum of GO and rGO, a sharp decrease in hump at 286.6 eV is observed, which signify reduction (corresponding to the removal of functional groups attached to carbon skeleton). Moreover, the peak at 285.8 eV (-OH group) is not present in rGO C1s spectra and the area under the peaks corresponding to the oxygen functional groups such as –C–O– and –O–C–O– (at 286.3 eV and 288.3 eV, respectively) decreases. Simultaneously, the area under the peak at 284.1 eV and 284.9 eV (–C=C– and –C–C–) increases in comparison to C1s spectra of GO (for details about fractional areas under the peaks, see Table S2 in SI) after exposure of UV light (2 h). Finally larger intensity corresponding to the –C=C– peak compared to that for –C–C– peak confirms the significant sp^2^ character of the rGO sample. Effects of longer exposures to UV lights (with different wavelengths and intensity/power) deserves further explorations.

A comparison of our method for GO synthesis with the popular KMnO_4_ based methods existing in literature is summarized in Fig. S3 (see SI). Table S3 (see SI) summarizes the yields. We can observe that the yield in our method is better than the other popular methods reported in literature. Using suitable tuning of reaction parameters including the PCP protocol, elevated reaction temperature, increased reaction time and a reduced H_2_O:H_2_O_2_ ratio during reaction termination, we are able to remove the requirement for expensive filtration membranes without compromising on the quality and yield of GO. We have also completely eliminated the explosive nature of the synthesis process, a major obstacle in scalable applications of GO (see supplementary videos)^31^.

**Figure 7:**
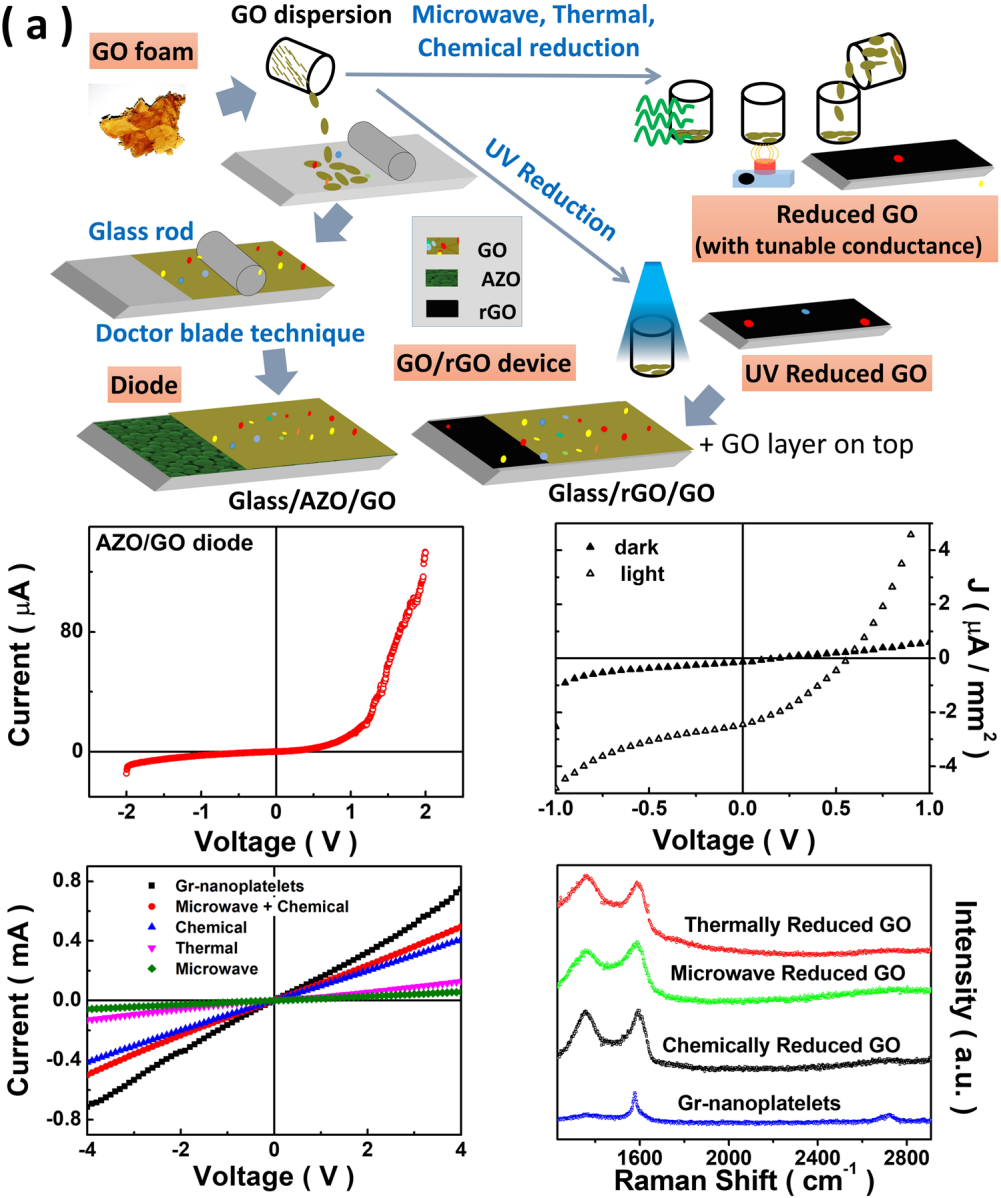
(**a**) Schematic diagram summarizing the typical applications demonstrated using the as synthesized GO material (dried GO foam). The actual optical image of a piece of dried GO foam obtained after vacuum desiccation of the as grown GO sample is also shown within the schematic. The GO films deposited on AZO is used for studying diode like behavior while the photovoltaic property is explored in the GO-rGO bilayer. Ultra-violet, microwave, thermal and chemical reduction protocols are used for making rGO samples. (**b**) Current-Voltage (I-V) characteristics of the GO-AZO bilayer. (**c**) Current-Voltage (I-V) characteristics of the GO-rGO bilayer in dark and upon exposure to 1.5AM Global sunlight. (**d**) Current-Voltage (I-V) characteristics of drop-casted films of rGO samples made using different reduction techniques. For a comparison, the I-V characteristics of a drop-casted film of commercially available graphene nanoplatelets is also shown. (**e**) Raman spectra for graphene nanoplatelet sample along with rGO samples made using different protocols.

We made use of the dried GO foam for film fabrications, reduction and a number of additional measurements in order to elucidate the utility of GO synthesized using our approach for certain applications. Figure 7(a) summarizes in brief the applications of GO demonstrated in this paper which include, diode behavior of AZO-GO multi-layer and the photovoltaic response of GO-rGO multi-layer. Conventional doctor’s blade technique^59^ was used to make the films of GO on ordinary glass and AZO coated glass substrates. Partial reduction of the film coated on glass was performed by following the identical reduction protocol as used for Fig. 5(a). Here, a UV exposure time of 2 hour was used to obtain the rGO in making the GO-rGO multi-layer on glass. Current-Voltage (I-V) characteristics across the GO-AZO and GO-rGO structures are shown in Fig. 7(b,c), respectively. A diode like I-V characteristics is observed in both the cases. As AZO is a n-type material, the above observation suggests the p-type nature of GO. Upon illumination with a 1.5 AM Global sunlight, a photovoltaic response is observed in the GO-rGO multi-layer with an open circuit voltage (V_*OC*_) of 0.6 V and a short-circuit current (I_*SC*_) of 3.6 *μ*A. A review of the existing literature on GO clearly demonstrates its importance as an anode buffer layer^76,77^ and hole transport material^78^ in organic solar cell since it allows holes to effectively travel towards the electrode and also hinders the electron transport. This leads to a reduction in the recombination of electron and hole pairs which results in improved device performance. Similarly rGO has been used as electron extraction material in perovskite solar cells^79^ but the demonstration of solar cells made up of all graphene layers by making a multi-layer structure of GO and rGO films has not been reported so far. We report for the first time a solar cell made up of all graphene based layers. More so, the solar cell demonstrated is fabricated through a cost effective technique comprising doctor blade technique for film deposition and inexpensive UV reduction for making rGO. This paves way for further work in this area for realizing practical efficiencies in near future.

We employ a host of reduction protocols to obtain rGO samples with different degrees of reduction^24,60,61^. Specifically, thermal reduction was carried out at 300 °C for 10 minutes and the microwave reduction was carried out using a conventional microwave oven operated at 800 W for 2 s. The chemical reduction protocol employed the green reduction of GO using L-ascorbic acid as proposed by Abdolhosseinzadeh *et al*.^60^. In Fig. 7(d), we show the I-V characteristics of drop-casted films for these rGO samples. For a comparison, the I-V characteristics of a drop-casted film (using identical dilution and drop volume) of commercially available graphene nanoplatelets (CAS 7782–42–5 from TCI) is shown in Fig. 7(d). The comparative Raman spectra for these different rGO samples is summarized in Fig. 7(e) along with the Raman spectra of graphene nanoplatelets. One can clearly observe that rGO samples with tunable conductance can be very conveniently made using high quality GO samples made using our method opening up possibility for a host of end applications. It is worth mentioning here that Abdolhosseinzadeh *et al*.^60^ did not perform the electrical characterization of reduced films and the starting GO used by them was made using an extensively time consuming process to avoid any explosions. Furthermore, the GO samples reported by Abdolhosseinzadeh *et al*.^60^ contained prominent amounts of Mn-based impurities. As can be observed from the inset in Fig. 6(a), we did not observe any signatures corresponding to Mn 2p_1/2_ and 2p_3/2_ peaks in the window 635–660 eV in the XPS measurements. For a comparison, one can look at the existence of distinct Mn 2p_1/2_ and 2p_3/2_ peaks in the range 635–660 eV of binding energies in Mn-containing samples (see Fig. 2(b) of Zhang *et al*.^80^). An optical image of a piece of dried GO foam obtained after vacuum desiccation of the as grown GO sample can be seen as part of the schematic (see Fig. 7(a)). Such GO foam has tremendous potential for bio-medical^1–4,6–8,10^ and energy storage applications^21,22,28^. Akin to the feasibility of scalability, the GO made using our method also has tremendous potential for environmental remediation applications^13–15^.

**Figure 8:**
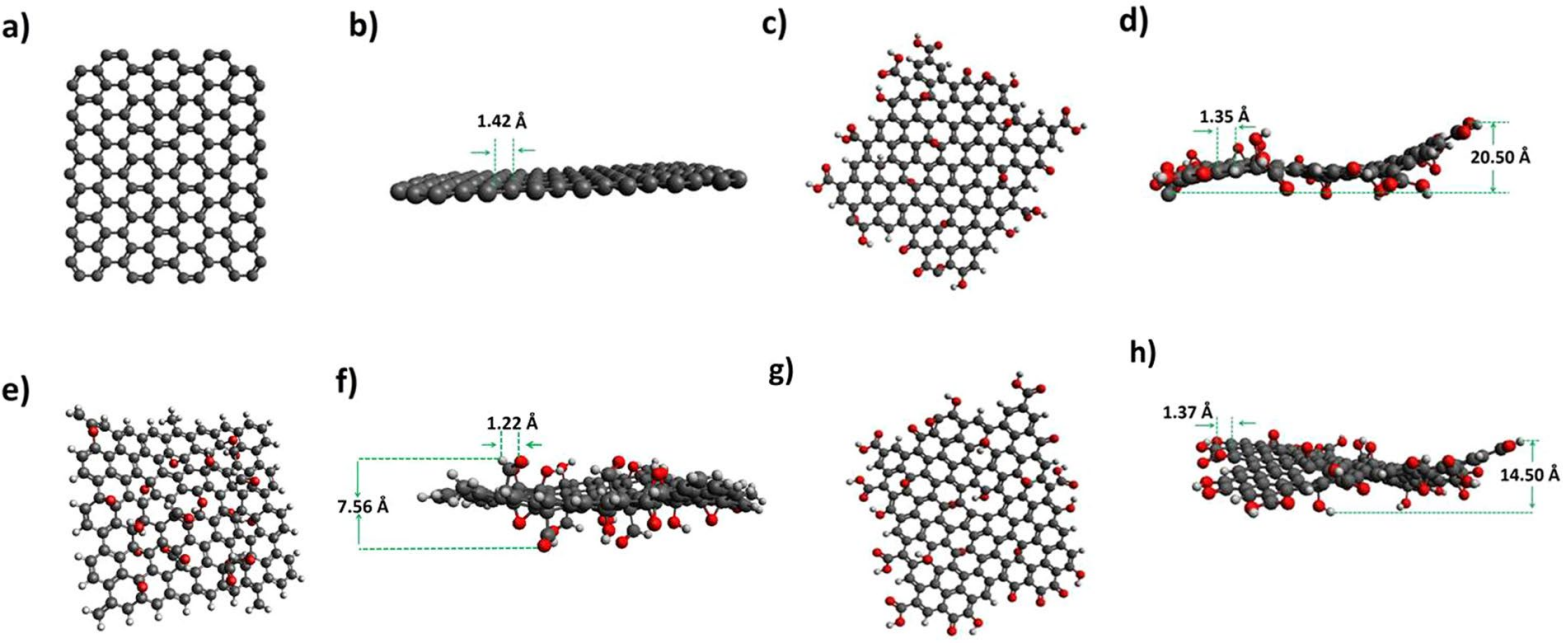
Optimized geometries for a 7 × 7 nanoflakes of graphene (top view (**a**); side view (**b**)), GO with comparable number of functional groups along the edges as well as in the basal plane (top view (**c**); side view (**d**)), GO with functional groups primarily in the basal plane and edge passivation using H (top view (**e**); side view (**f**)), rGO with a number of functional groups removed from the GO nanoflake with functional groups along the edges as well as in the basal plane (top view (**g**); side view (**h**)). Various distances are marked to provide an idea regarding the wrinkles.

Using high-resolution solid-state ^13^C-NMR, Cai *et al*.^81^ confirmed the existence of C–O–C, sp^2^ C and **C–OH** in GO. We also found evidence for these in our samples based on FTIR and XPS measurements. A one to one connection between removal of **C–OH** groups and an enhancement in conductivity is also corroborated from our data. To gain additional understanding about the synthesized GO samples, we performed certain density functional theory (DFT) calculations. DFT techniques have been extensively used in past to understand various aspects of GO physics^82–87^. For our purpose, we constructed a structural model for GO comprising of a carbon backbone in the form of a single graphene sheet to which various functional groups were attached. A 7 × 7 chain of carbon honeycomb structure was used and we incorporated four ketonic group at the surface, seven carboxylic group at edges, four aldehyde group at the edges and twelve hydrogen atoms at edges for creating a realistic model of GO (labeled GO-A). We considered another 7 × 7 chain of carbon honeycomb structure with a larger number of epoxides and hydroxyl functional groups attached primarily in the basal plane and the edge was passivated using H (GO-B). For comparison, we also constructed 7 × 7 structural models for graphene (labeled G) and rGO. In case of rGO, we removed a fraction of functional groups from the structural model of GO-A above. Mono-vacancy defect site^88^ has also been introduced in the structural model of rGO (labeled rGO-A). Structural optimization using DFT was done utilizing Gaussian-16. The calculations were performed with M062X using 6–31 G* basis set on supermicro SYS-6028R-TR server with two octa-core Intel(R) Xenon(R) CPU E5-2620 v3-2.40 GHz processors^89,90^. It took about 60 h for the structure optimization of 7 × 7 GO using all the 16 CPUs of the server. On the other hand, the structure optimization for the 7 × 7 rGO and graphene took 38 h and 3 h, respectively. The stabilized structures for graphene, GO and rGO for our starting structural configurations are shown in Fig. 8.

DFT is also capable of providing useful spectroscopic information^91^. Here, we attempt to reconstruct the Raman spectrum of GO for the structural model proposed above employing the technique of time-dependent density functional theory (TD-DFT) using Guassian 16. For this, we used MO6-2X functional and 631-G* as basis set. The results indicate the presence of bands at 1385 cm^−1^, 1615 cm^−1^ and 2852 cm^−1^ (see SI, in particular Fig. S4 for further details), which corresponds to D, G and 2D bands of GO, respectively (see Fig. S4 and main panel of Fig. 2(a) for comparison). Further work is desirable to establish the atomic configurations and related electronic properties in GO^92^.

## Conclusions

To conclude, we present an inexpensive route for synthesizing GO via modifications to Hummers’ approach^50–53^. The key modifications involve the PCP protocol in addition to crucial changes in reaction time and temperature and the concentration of reaction terminating agent (H_2_O_2_ in this case). The proposed synthesis approach completely eliminates the explosive nature of the underlying reactions. Using this approach we are able to obtain high yields of very good quality GO as exemplified by XRD, electron microscopy, FTIR, Raman spectroscopy and XPS results presented in this work. Using DFT and TD-TDFT, we have looked at a simple structural model for monolayer GO and rGO and a striking reconstruction of observed Raman data is seen. It should be noted here that our approach does not require the use of expensive filtration membranes^53^. A simple reduction protocol based on UV exposure is also demonstrated to make rGO from GO. Observation of diodic behavior in an AZO-GO multi-layer attests to the p-type character of GO. The possibility of using a GO-rGO multi-layer for energy harvesting based on photovoltaic response has not gone unnoticed. Due to inexpensive and scalable synthesis method, applications requiring GO dispersion, e.g., dye adsorption for environmental remediation is also feasible for large scale end applications^11–16^. GO foam obtained using our method can be used for bio-medical^1–4,6–8,10^ and energy storage applications^21,22,28^. The absence of toxic gas emission coupled with the inexpensive nature of the synthesis technique has the potential to open the flood-gates for large scale applications of GO in domains including electronics, bio-medicine, energy and environment.

## Methods

All chemicals used in synthesis process were purchased from commercial source (Sigma Aldrich and CDH) and used without further purification. In our strategy we used graphite flakes (C), sulphuric acid (H_2_SO_4_), phosphoric acid (H_3_PO_4_), hydrochloric acid (HCl), hydrogen peroxide (HCl), deionized (DI) water and potassium permanganate (KMnO_4_). Our method is adapted from Marcano’s method^53^ with certain crucial modifications. Initially graphite flakes and potassium permanganate (in the ratio 1:6) were mixed in a mortar and pestle for 5 minutes and kept for pre-cooling to 5 °C (the pre-cooling protocol, PCP). A separate solution of sulphuric acid and phosphoric acid (in the ratio 9:1) was prepared and also pre-cooled to 5 °C. The acid solution was then added to the mixture of graphite flakes and potassium permanganate with continuous stirring (using a magnetic stirrer) which makes the color of the solution as greenish black (see Fig. S1(a)). The temperature does not exceed room temperatures at this step despite the exothermic nature of the reaction. The solution so obtained was heated at 65 °C and was left for 24 h with continuous stirring. After 24 h the solution becomes brownish in color (see Fig. S1(b)) and was allowed to cool till room temperature is achieved. This is then added to a beaker containing 400 ml deionized water ice (see Fig. S1(c)). After this 7 ml of H_2_O_2_ was added to it while stirring with a glass rod. The solution color changes to golden yellow (see Fig. S1(d)), marking the formation of GO. Distilled water was added to the solution after 5 minutes and then the precipitate was allowed to settle down for an hour. This is followed by successive washing with DI water (3 times), HCl (2 times), ethanol (3 times) and DI water (3 times) with intermediate centrifugation (at 10,000 rpm for 5 minutes) and decantation. The gel thus obtained was degassed for 24 hours in a desiccator leading to the formation of GO foam. GO dispersion in a solvent (say water) can be prepared by dispersing either the as synthesized gel or the GO foam in the solvent followed by ultrasonication. Films of GO can be made from GO dispersion using any of the conventional techniques including spin-coating, drop-casting, dip-coating, spray-painting and doctors blade technique^59^. For the characterization of our samples, following equipment were utilized: (a) Rigaku TTRX-III diffractometer with 1.54 Å Cu-K_*α*_ X-ray source for X-ray diffraction, (b) confocal micro-Raman spectrometer from Seki Technotron corporation, Japan with 514.5 nm Argon ion laser for Raman studies, (c) Shimadzu IrAffinity-1 for FTIR spectroscopy, (d) Perkin Elmer Lambda-35 UV-Vis spectrophotometer for UV-Vis spectroscopy, (e) LEO 435 VP SEM for SEM imaging, (f) Jeol, JEM 2100 for obtaining TEM images and SAED patterns, (g) ESCA + Omicron Nano Technology GmbH for XPS studies, (h) Photo Emission Tech solar simulator model SS50AAA-EM with illumination area of 50 mm × 50 mm and Air Mass Filter of AM1.5 Global, and (i) Keithley source-meter 2420 for electrical characterization.

## Electronic supplementary material

Supplementary Information file

Step1_synthesis

Step2_synthesis

Step3_synthesis

Step4_synthesis

Dataset ALL
