# Author Correction: A Low-Cost Non-explosive Synthesis of Graphene Oxide for Scalable Applications

**DOI:** 10.1038/s41598-018-32556-2

**Published:** 2018-09-26

**Authors:** Pranay Ranjan, Shweta Agrawal, Apurva Sinha, T. Rajagopala Rao, Jayakumar Balakrishnan, Ajay D. Thakur

**Affiliations:** 1 Department of Physics, Indian Institute of Technology Patna, Bihta, 801106 India; 2 Department of Chemistry, Indian Institute of Technology Patna, Bihta, 801106 India; 3 Department of Physics, Indian Institute of Technology Palakkad, Palakkad, 678557 India

Correction to: *Scientific Reports* 10.1038/s41598-018-30613-4, published online 13 August 2018

This Article contains errors.

In panels (b) and (c) of Figure 6, ‘O-C-O/C=O’ is incorrectly given as ‘O-C-O’. The correct Figure 6 appears below as Figure 1.

**Figure 1:**
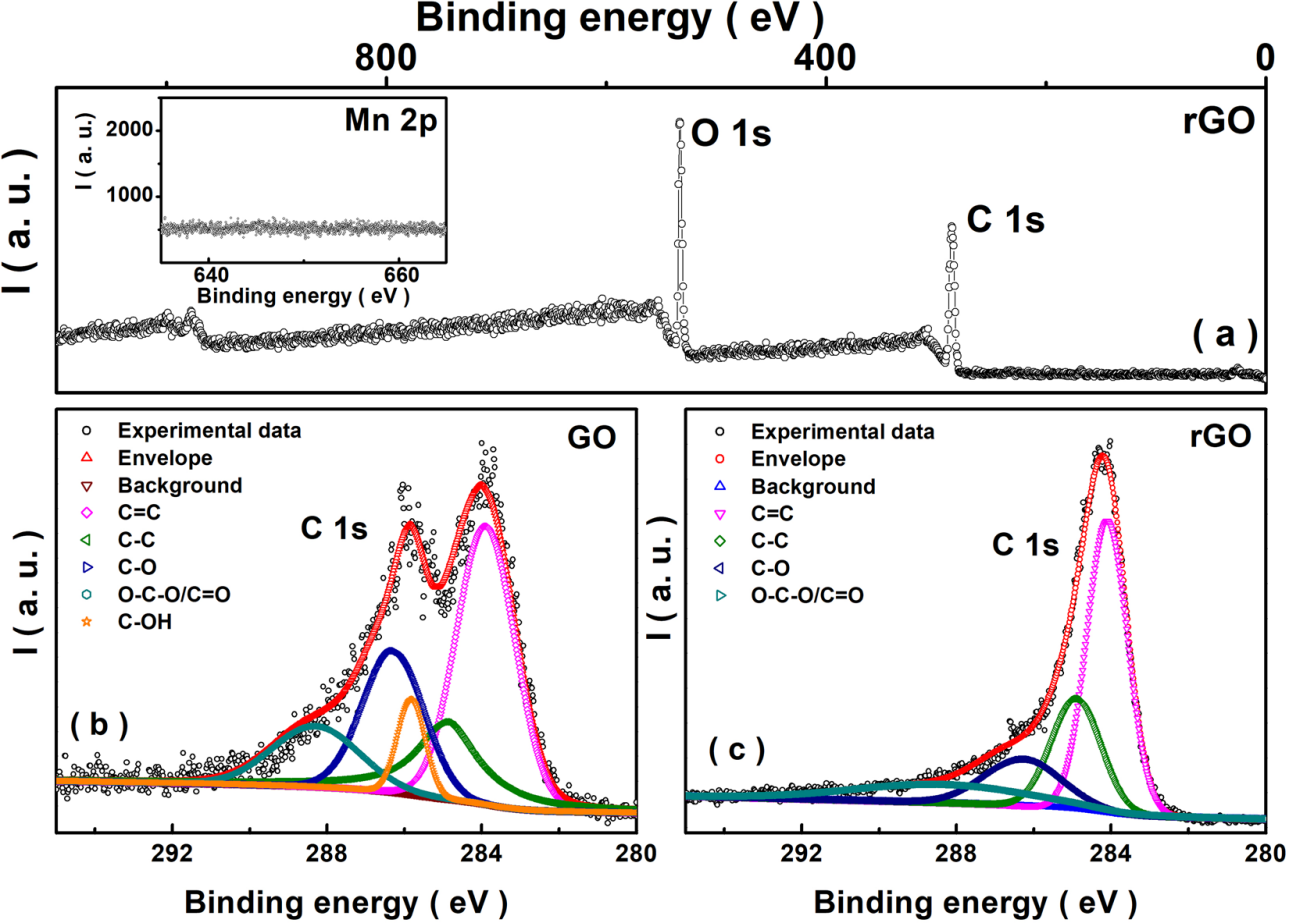
(**a**) Intensity (I) versus binding energy (B. E.) in the full scan range for the XPS of the rGO film obtained after a 2 hour exposure to a 4 Watt, 365 nm UV lamp. (**b**) A multiple peak deconvolution of the GO C1s XPS data (corresponding to –C–C–, –C=C–, –C–O, –O–C–O–, –OH and –C=O respectively). The inset in panel (a) shows the XPS data corresponding to Mn 2p binding energy region for the rGO sample. (**c**) rGO C1s XPS data for the sample along with the deconvoluted peak structure corresponding to –C–C–, –C=C–, –C–O, –O–C–O–, and –C=O are marked.

In addition, Supplementary Table S2 contains the following errors.

“-C=O”

should read:

“C-O”

and

“C-O-C”

should read:

“O-C-O/C=O”

