# Supplementary material for: A Low-Cost Non-explosive Synthesis of Graphene Oxide for Scalable Applications
Source: Sci Rep. 2018 Aug 13;8:12007. doi: 10.1038/s41598-018-30613-4 (PMC6089993; doi:10.1038/s41598-018-30613-4)
Supplement: Supplementary file 1 — Supplementary Information file [file 41598_2018_30613_MOESM1_ESM.pdf]

Supplementary material: A Low-Cost Non-explosive Synthesis of Graphene Oxide for Scalable Applications

Pranay Ranjan[1,*], **Shweta Agrawal**[2], **Apurva Sinha**[1], **T. Rajagopala Rao**[2], **Jayakumar Balakrishnan**[1,3], **Ajay D. Thakur**[1,*]

[1]Department of Physics, Indian Institute of Technology Patna, Bihta 801106 INDIA
[2]Department of Chemistry, Indian Institute of Technology Patna, Bihta 801106 INDIA
[3]Department of Physics, Indian Institute of Technology Palakkad, Palakkad 678557 INDIA
[*]Correspondence and requests for materials should be addressed to P.R. (email: pranjan@iitp.ac.in) or A.D.T. (email: ajay.thakur@iitp.ac.in)

We provide below the supplementary information associated with the manuscript. It comprises of four figures and three tables. Figure 1 shows optical images of the reaction beaker at various stages of the reaction, viz., (a) after mixing the reactants at the beginning of the process, (b) prior to the addition of hydrogen peroxide, (c) when hydrogen peroxide is added, and (d) after the reaction is complete. Figure 2 shows the x-ray diffraction(XRD) spectrum of GO samples prepared at various time and temperature along with XRD spectra of the starting precursor. International center for diffraction data (ICDD) card number of carbon and graphite are marked. Figure 3 is a schematic diagram which shows a comparison of our approach with the existing methods for the synthesis of GO using $KMnO_4$ as the oxidizing agent pointing out the key differences. This is further elaborated in Table S1[1–9]. Figure 4 shows the time-dependent density functional theory (TD-DFT) Raman spectra of monolayer GO (7×7) obtained using Guassian-16 with inset showing the optimized structure (side and top views) used for GO: (a) with comparable number of functional groups along the edges as well as in the basal plane, (b) with functional groups primarily in the basal plane and edge passivation using H. The dominant vibrational modes in 1 layer graphene nanoflake (GNF) are D, G and 2D bands with some additional overtone and combination modes such as D+D$'$, G+D$''$, D+D$''$, etc[10]. Out of these, the 2D and D+D$''$ arises due to the triple resonance (TR) or, double resonance (DR) processes and depends on the electronic properties of the GNF. Raman spectra of 1 layer GNF contain peaks in the spectral region between 1500 cm^{-1} to 3400 cm^{-1}. The G band corresponds to E_{2g} vibrational mode and results from the in plane vibration of carbon atoms. Except G band, the appearance of 2D in the spectral range of 2700 cm^{-1} to 3240 cm^{-1} involves overtone of transverse optic (TO) phonon at K point activated by triple resonance Raman scattering (TRRS). Evolution of 2D$'$ mode is attributed to the activation of TRRS mode by overtone of longitudinal optic (LO) phonon mode at Γ point. In addition to this, disappearance of D and D$'$ mode (fundamental to 2D and 2D$'$ mode) in 1 layer GNF is due to absence of defects in GNF. However, D and D$'$ modes are present in defect induced GNF and has also been observed in our case as we explored the addition of functional groups in the basal plane in the system. It should be noted that appearance of D band in 1 layer GNF is expected as edges are a kind of defect leading to breaking of translational symmetry. Thus, D and D$'$ may also arise in 1 layer GNF. Moreover, the presence of lower order Raman peaks in TD-DFT simulated GNF structures is attributed to acoustic mode or a combination of acoustic modes such as the transverse acoustic (TA) $\sim$ 220 cm^{-1}, longitudinal acoustic (LA) $\sim$ 350 cm^{-1}, 2TA $\sim$ 430 cm^{-1}, 2LA $\sim$ 690 cm^{-1}, TA+LA $\sim$ 570 cm^{-1}, TA+D$'$ $\sim$ 1830 cm^{-1}, LA+D$'$ $\sim$ 1940 cm^{-1}, etc.[10–12]. TD-DFT is a useful method capable of providing useful spectroscopic information[12]. Here, we attempt to reconstruct the Raman spectrum of GO for the structural models shown in the inset of Fig. S4 (a) and Fig. S4 (b) employing the technique of TD-DFT using Guassian-16. For this, we used MO6-2X functional and 631-G* as basis set. We have used different degree of oxidized GO nanoflakes (GONFs) and compared its Raman spectra with edge passivated graphene sheets. We observe D, G and 2D band at 1385 cm^{-1}, 1615 cm^{-1} and 2852 cm^{-1} for GONF (panel (a) of Fig. S4). For another GONF with a different degree of oxidation due to more number of oxygen functional group in the basal plane, we observe bands at 1200 cm^{-1}, 1538 cm^{-1} and 3228 cm^{-1} corresponds to D, G and 2D bands respectively (see Fig. S4 and main panel of Fig 2 (a) for comparison). For the GONF in the inset of Fig. 4(a), the I_D/I_G ratio is found to be 0.96, whereas for the GONF in Fig. S4 (b), we observe an I_D/I_G ratio of 0.66. While, further work is desirable to establish the connection between atomic configurations and related electronic properties in GONF, one can observe that TD-DFT results for the proposed structural models agree well with the experimentally observed Raman data (see Fig. 2(a)). Table S2 provides a comparison of fractional areas under various deconvoluted peaks in the C 1s XPS spectra of GO and rGO (as shown in Fig. 6 of the main manuscript). Table S3 provides a comparison of yield in our method with other popular methods for synthesis of GO.

We used doctor blade technique[13] to deposit the films used in the fabrication of the diode and photovoltaic device (as reported in Fig. 7 of the main manuscript). A host of reduction techniques[14–16] and their specific combinations have been used in the preparation of rGO samples reported in this work. Specifically, thermal reduction was carried out at 300°C for 10 minutes and the microwave reduction was carried out using a conventional microwave oven operated at 800 W for 2 s. The chemical reduction protocol employed the green reduction of GO using L-ascorbic acid as proposed by Abdolhosseinzadeh *et*

al[14]. It is worth mentioning here that Abdolhosseinzadeh *et al*[14] did not perform the electrical characterization of reduced films and the starting GO used by them was made using an extensively time consuming process to avoid any explosions.

References

1. Brodie, B. C. On the atomic weight of graphite. *Phil. Trans. R. Soc. Lond.* **149**, 249–259 (1859).
2. Staudenmaier, L. Verfahren zur Darstellung der Graphitsäure. *Ber. Dtsch. Chem. Ges.* **31**, 1481–1487 (1898).
3. Hofmann, U. *Ü*ber Graphits*ä*ure und die bei ihrer Zersetzung entstehenden Kohlenstoffarten. *Ber. Dtsch. Chem. Ges.* **61(2)**, 435-441 (1928).
4. Hummers Jr. W. S., Offeman, R. E. Preparation of graphitic oxide. *J. Am. Chem. Soc.* **80(6)**, 1339–1339 (1958).
5. Kovtyukhova, N. I. et al. Layer-by-layer assembly of ultrathin composite films from micron-sized graphite oxide sheets and polycations. *Chem. Mater.* **11**, 771–778 (1999).
6. Hirata, M., Gotou, T., Horiuchi, S., Fujiwara, M., Ohba, M. Thin-film particles of graphite oxide 1: High -Yield synthesis and flexibility of the particles. *Carbon* **42**, 2929–2937 (2004).
7. Marcano, D. C. et al. Improved synthesis of graphene oxide. *ACS Nano* **4**, 4806–4814 (2010).
8. Marcano, D. C. et al. Correction to improved synthesis of graphene oxide. *ACS Nano* **12(2)**, 2078–2078 (2018).
9. Peng, Li. An iron-based green approach to 1-h production of single-layer graphene oxide. *Nat. Commun.* **6**, 5716 (2015).
10. Wu, J.-B., Lin, M.-L., Cong, X., Liu, H.-N., Tan, P.-H., Raman spectroscopy of graphene-based materials and its applications in related devices. *Chem. Soc. Rev.* **47**, 1822 (2018).
11. Krishnamoorthy, K., Veerapandian, M., Yun, K., Kim, S.-J. The chemical and structural analysis of graphene oxide with different degrees of oxidation. *Carbon* **53**, 38-49 (2013).
12. Zhang, W., Carravetta, V., Li, Z., Luo, Y., Yang, J. Oxidation states of graphene: Insights from computational spectroscopy. *J. Chem. Phys.* **131**, 244505 (2009).
13. Berni, A., Mennig, M., Schmidt, H. Sol-Gel Technologies for Glass Producers and Users. 89-92 (Springer Science + Business Media, New York (2004) DOI 10.1007/978-0-387-88953-5).
14. Abdolhosseinzadeh, S., Asgharzadeh, H. Kim, H. S, Fast and fully scalable synthesis of reduced graphene oxide. *Scientific reports* **5**, 10160 (2015).
15. Kumar, P. V. New insights into the thermal reduction of graphene oxide: Impact of oxygen clustering. *Carbon* **100**, 90-98 (2016).
16. Voiry, D. et al. High quality graphene via microwave reduction of solution exfoliated graphene oxide. *Science* **353(6306)**, 1413-1416 (2016).

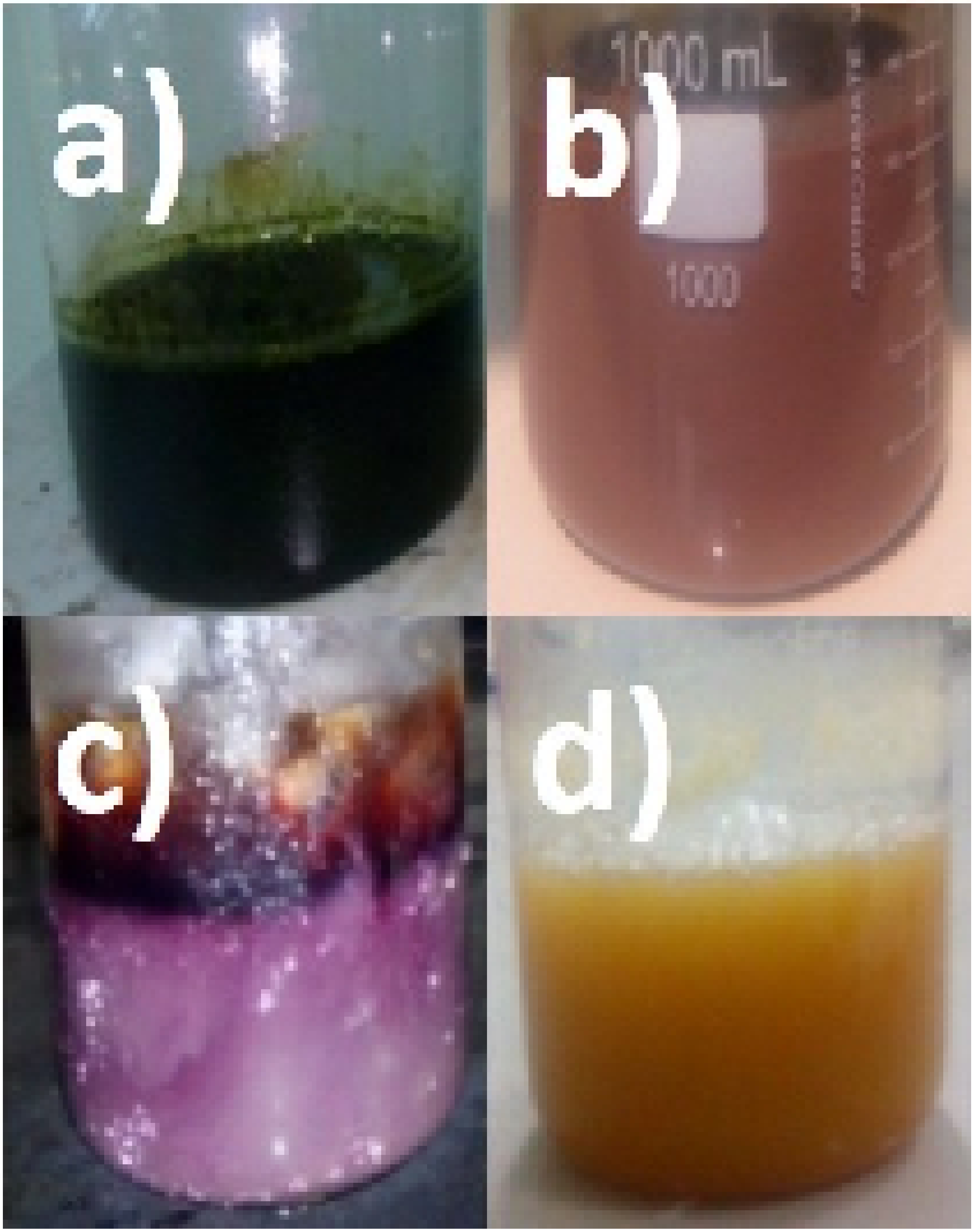

Figure S1. Images captured at various stages of reaction: (a) after mixing the reactants at the beginning of the process, (b) prior to the addition of hydrogen peroxide, (c) when hydrogen peroxide is added, and (d) after the reaction is complete.

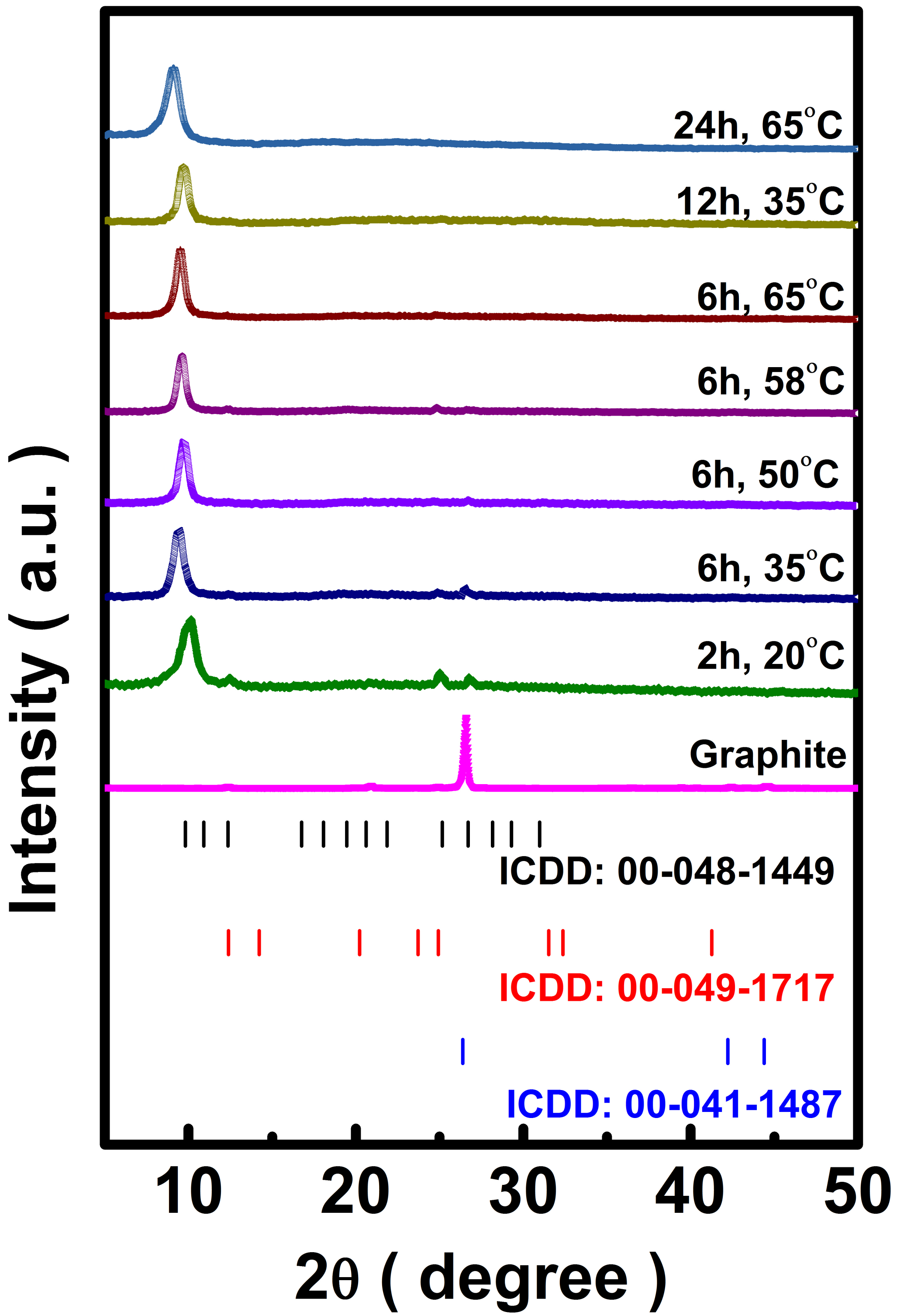

Figure S2. XRD spectrum of GO samples prepared at various time and temperature along with starting precursor XRD spectra. ICDD card number of carbon and graphite are marked.

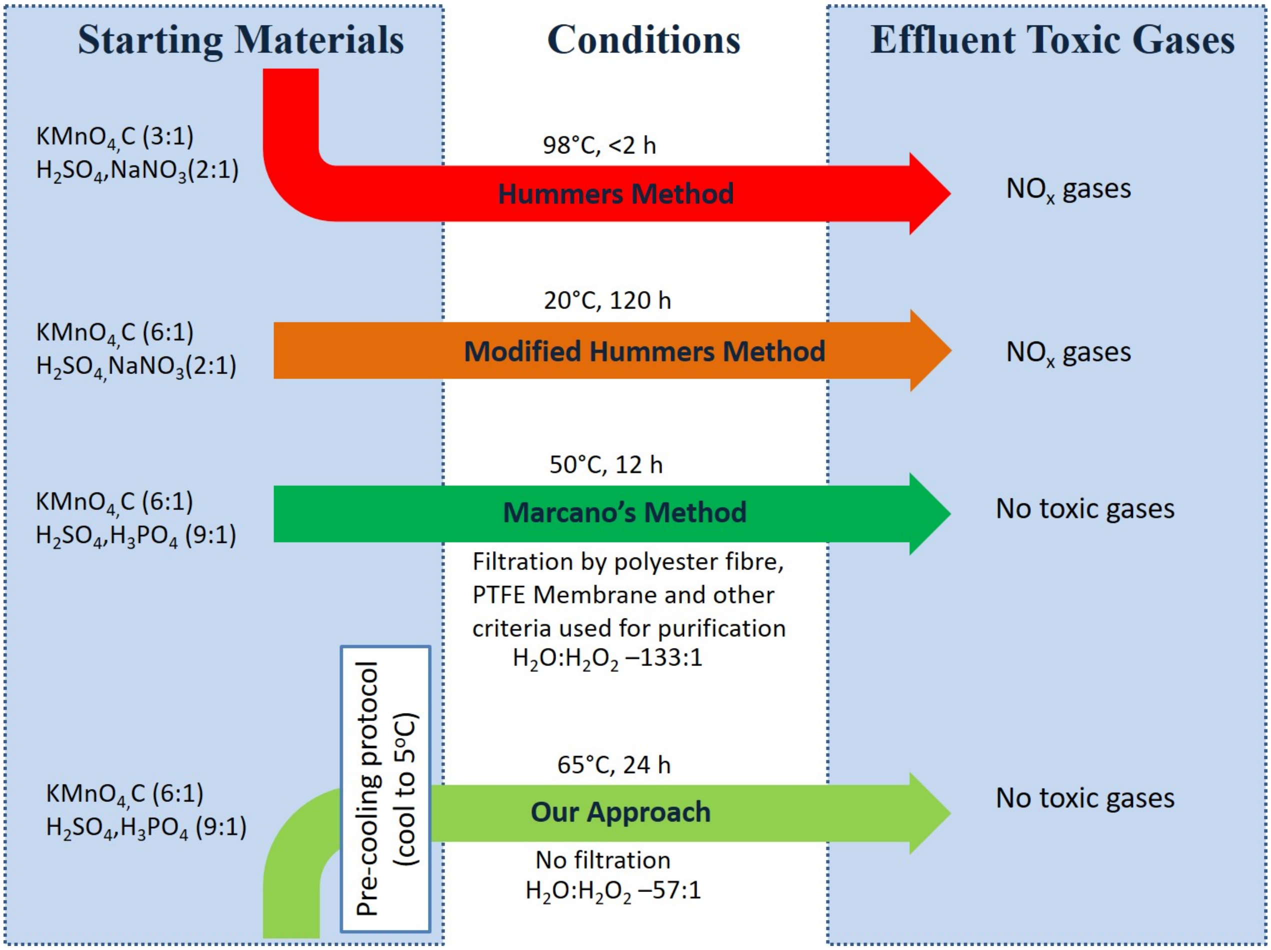

Figure S3. Comparison of our approach with the existing methods for the synthesis of GO using $KMnO_4$ as the oxidizing agent.

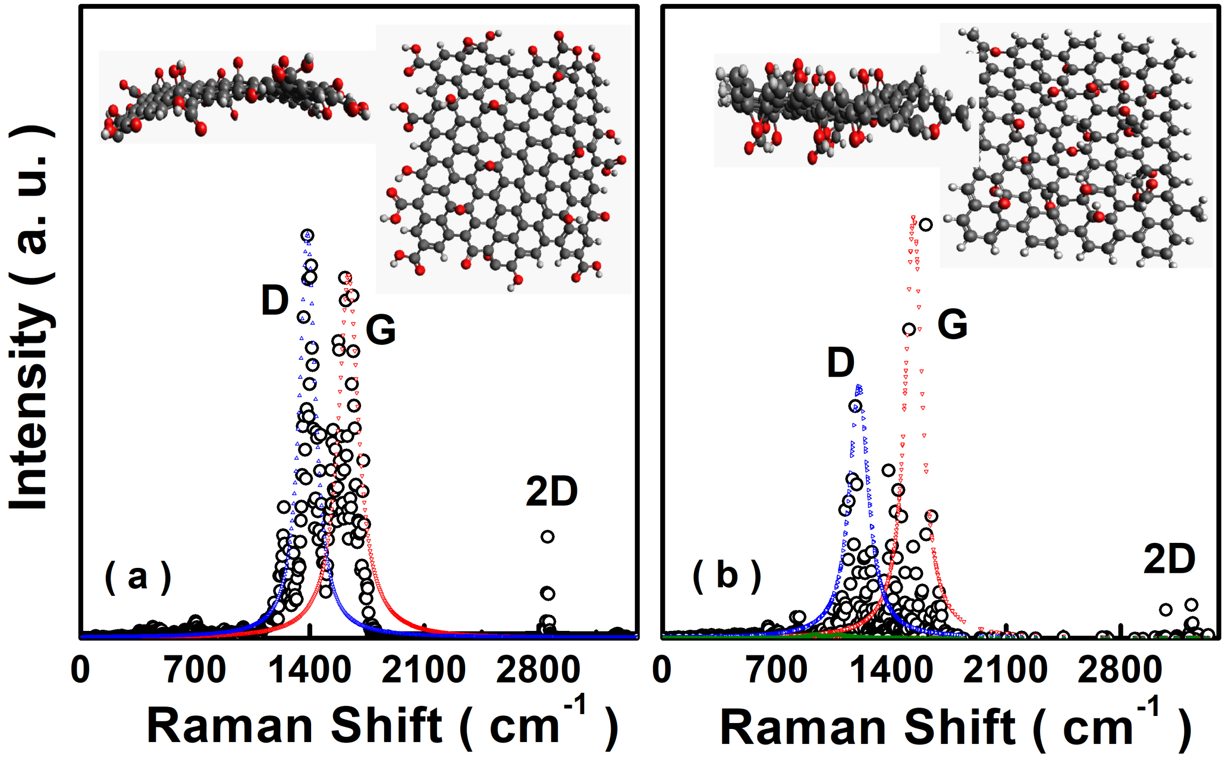

Figure S4. Monolayer GO (7×7) TD-DFT Raman spectra obtained using Guassian-16 with inset showing the optimized structure (side and top views) used for GO: (a) with comparable number of functional groups along the edges as well as in the basal plane (GO-A), (b) with larger number of epoxides and hydroxyl functional groups primarily in the basal plane and edge passivation using H (GO-B).

Table S1. Comparison of various methods for synthesizing GO.

Method (year)	Nature of Oxidant	Reaction Time	Interlayer Spacing	Toxic Gases	Residues	Degree of Hazard
Brodie (1859)[1]	$KClO_3$ based	10h	5.95Å	ClO_2	$KClO_3$	Explosive
Staudenmaier (1898)[2]	$KClO_3$ based	1-10 days	6.23Å	ClO_2, NOx	$KClO_3$	Explosive
Hofmann (1937)[3]	$KClO_3$ based	4 days	-	ClO_2, NOx	$KClO_3$	Explosive
Hummers (1958)[4]	$KMnO_4$ based	2-10 h	6.67Å	NOx	Mn_2O_7	Mild explosive
Modified Hummers-I (1999)[5]	$KMnO_4$ based	8 h	6.9Å	-	Mn_2O_7	Explosive
Modified Hummers-II (2004)[6]	$KMnO_4$ based	5 days	8.3Å	NOx	Mn_2O_7	Mild explosive
Modified Hummers-III (2010)[7]	$KMnO_4$ based	12 h	9.3Å	-	-	Non-explosive[8]
Peng's method (2015)[9]	K_2FeO_4 based	1 h	9.0Å	No toxic gases	Fe^{+3}	-
Our method (2016)	$KMnO_4$ based	24 h	9.74Å	No toxic gases	not found	Non-explosive

Table S2. Comparison of fractional areas under various deconvoluted peaks in the C 1s XPS spectra of GO and rGO.

Sample	Fractional area under peak					sp^3 (%)
	283.9/284.1 eV (C=C)	284.9 eV (C-C)	285.8 eV (-C-OH)	286.3 eV (-C=O)	288.3 eV (C-O-C)	
GO	0.406	0.176	0.066	0.218	0.134	59.4%
rGO	0.490	0.227	-	0.149	0.134	51%

Table S3. Comparison of yield through various methods for synthesis of GO.

Sl. No.	Reaction Time, Temperature	Initial Wt.	Final Wt.	Reference
1	24h, 65°C	3 gm	6.49 gm	Our Work
2	12h, 50°C	3 gm	5.8 gm	Marcano's method[7,8]
3	15 min, 98°C	3 gm	1.2 gm	Hummers' method[4,7]
4	12h, 35°C	3 gm	4.2 gm	Modified Hummers' method[6,7]
